# Development of database structure and indexing for siddha medicine system – A platform for siddha literature analytics

**DOI:** 10.1016/j.dialog.2022.100008

**Published:** 2022-05-05

**Authors:** Dhivya Karmegam, Muthuperumal Prakash, N. Karikalan, Bagavandas Mappillairajan

**Affiliations:** a School of Public Health, SRM University, Kattankulathur 603 203, Kancheepuram District, Tamil Nadu, India; b Centre for statistics, SRM University, Kattankulathur 603 203, Kancheepuram District, Tamil Nadu, India; c National Institute for Research in Tuberculosis, Chetpet, Chennai 600 031, Tamil Nadu, India

**Keywords:** Traditional medicine, Database management system, Siddha, MySQL workbench, Machine learning

## Abstract

**Background:**

Siddha Medicine system is one among the oldest traditional systems of medicines in India and has its entire literature in the Tamil language in the form of poems (*padal* in tamil). Even if the siddha poems are available in public domain, they are not known to other parts of the world because, researchers of other languages are not able to understand the contents of these poems and there exists a language barrier. Hence there is a need to develop a system to extract structured information from these texts to facilitate searching, comparing, analysis and implementing.

**Objective:**

This study aimed at creating a comprehensive digital database system that systematically stores information from classical Siddha poems and to develop a web portal to facilitate information retrieval for comparative and logical analysis of Siddha content.

**Methods:**

We developed an expert system for siddha (eSS) that can collect, annotate classical siddha text, and visualizes the pattern in siddha medical prescriptions (Siddha Formulations) that can be useful for exploration in this system using modern techniques like machine learning and artificial learning. eSS has the following three aspects: (1) extracting data from *Siddha* classical text (2) defining the annotation method and (3) visualizing the patterns in the medical prescriptions based on multiple factors mentioned in the Siddha system of medicine. The data from three books were extracted, annotated and integrated into the developed eSS database. The annotations were used for analyzing the pattern in the drug prescriptions as a pilot work.

**Results:**

Overall, 110 medicinal preparations from 2 *Siddhars* (*Agathiyar and Theran*) were extracted and annotated. The generated annotations were indexed into the data repository created in eSS. The system can compare and visualize individual and multiple prescriptions to generate a hypothesis for siddha practitioners and researchers.

**Conclusions:**

We propose an eSS framework using standard siddha terminologies created by WHO to have a standard expert system for siddha. This proof-of-concept work demonstrated that the database can effectively process and visualize data from siddha formulations which can help students, researchers from siddha and other various fields to expand their research in herbal medicines.

## Highlights

• Systematically documenting and retrieving information of siddha medicine system is the pioneering effort. Analytical exploration of the siddha content is never been done before.
• Siddha system of medicine has innumerable number of poems. We need to keep on updating the poems in the database.
• Webpage developed will be useful for knowledge discovery & analytical exploration of logic underlying drug formulations using taste and other factors

## 1 Introduction

The Indian system of medicines is one of the oldest traditional medicine systems which have a sound scientific background and a vast history of effectiveness and values [1]. The Indian System of Medicine holds six unique recognized systems practiced in India that includes Ayurveda, Yoga and Naturopathy, Unani, Siddha and Homoeopathy (AYUSH) [2,3]. These medicine systems are highly codified and available in public domain but still, they are unknown to other parts of the world because they are available only in local languages such as Sanskrit, Tamil, and Urdu. We aimed at breaking the language barrier of Siddha Medicine system, which is in the Tamil language in the form of poems by annotating and creating a structured database.

### 1.1 Siddha, a traditional medical system

Siddha, a traditional medical system of India and is one among the oldest traditional systems of medicines, taking root from South India around 5000 BC. It is of Dravidian origin and has its entire literature in the Tamil language. This system is being influenced by the local tradition with roots in the ancient Dravidian culture. Siddha system is considered as the scientific reflection of the intuitions of Siddhars (Ancient scholars). As per the siddha literature's there are eighteen Siddhars who lived in different periods in the southern part of India have prescribed their own medicinal prescriptions for various diseases and they are now available in the form of printed texts and palm leaf manuscripts. As Siddha is one of the earliest native forms of health practices, it is vital to preserve it efficiently and take it forward in the near future [4].

Siddha medicinal system is described in the form of poems. Each poem provides details like name of the disease, medicine name, combination and proportion of ingredients (minerals, herbs & animal products), method of medicine preparation, dos recommended, method of consuming the medicine, and period of medication. Since these texts are written in Tamil language, clinical and medical researchers of other languages and other regions are not able to understand the contents of these texts on their own and there exists a language barrier. Even for Tamil scholars annotation is needed to understand the contents of these poems [5]. Siddha System of Medicine emphases on considering and treating the root cause of the disease instead of treating the disease symptoms. Hence Siddha Medicine should not be considered as an alternative system of medicine alone, but also as a research tool to bring advancement in medicine [6]. Formulations indicated in Siddha literature centuries back are found to be factual, safe and effective in treatment [[7], [8], [9], [10]]. Even now in rural and tribal areas, Siddha healers play a vital role in providing primary healthcare [11].Many medicines for even for challenging diseases were discovered from traditional medicines by using the advancement in science like Reverse pharmacology and Network Pharmacology.

WHO Traditional medicine strategy (2002–2005) states that maintaining databases of information regarding traditional medicine will help in sharing scientific knowledge between focus groups and the public. Database Management Systems (DBMS) is the consistent way to store, organize and retrieve data with utmost efficiency. Database administrators and other specialists develop databases for various applications, like online business [12], cloud service [13], etc. Using the database to organize data, is highly recommended as it reduces the cost of storage media [14].

Databases like Traditional Chinese medicine, gene and Disease Information using Text mining (TCMGeneDIT) [15], TM-CTS [16], Traditional Chinese medicine (TCM) Database@Taiwan [17], Traditional Chinese Medicine Integrated Database (TCMID) [18], The Traditional Chinese medicine systems pharmacology (TCMSP) [19], Etc have been developed to store and retrieve Traditional information about Chinese Medicine for different determinations. The Central Council for Research in Ayurvedic Sciences (CCRAS), Government of India developed a database on medicinal plants used in Ayurveda and Siddha. Traditional Knowledge Digital Library (TKDL) has documented 12,000 Siddha Formulations [20]. But TKDL and other siddha databases don't capture all the information of Siddha poems completely and are limited to performing analytical exploration of the content. Hence developing a digital database and a web platform for analysis is an ideal solution to extract and use information from these poems for research purposes.

### 1.2 Objective

The objective of this study is

• To create a comprehensive digital database system that systematically stores and retrieves all possible information from classical Siddha poems.
• Also to develop a web portal to facilitate information retrieval for comparative and logical analysis for different end users including public, physicians, clinical and pharmaceutical researchers.

## 2 Methods

### 2.1 Design overview

The project necessitates bringing in people from various disciplines like Siddha, Tamil, Information Technology (IT) and Statistics. The role of Siddha and Tamil Scholars is to search, locate, provide Siddha poems, and also translate, explain and interpret the details in the poem. Database and algorithm development are done by IT Scholars, which is being explained in detail in this article.

The design concept for the database creation and indexing include 4 phases; they are Data Inventory, Database creation, Data retrieval, and analytics.

1. Data inventory includes collecting Siddha Medical scripts and the details about the scripts. Details include author and period of the scripts. Those details are helpful in indexing the Siddha poem and this is done by Siddha and Tamil scholars.
2. Database creation includes creating a library of Siddha words and its corresponding meaning and details as tables in MySQL.
3. Data retrieval includes creating a user interface to get the information from the poem based on the search criteria. Algorithm for the search engine is written to frame the query and search in the database.
4. In the analytic phase, the webpage is developed for the purpose of retrieving information to analyse the prescription consistency of drugs for the condition of disease by different authors (siddhars) and visualize it for easy understanding and exploration.

### 2.2 Database design

The database design is an important step as it will make the database efficient and effective in organizing large datasets. There are lots of DBMS systems available in the market from which one needs to be picked based on the effectiveness. MySQL is the free open source relational database management system which has been widely used by web developers. Even though MySQL is free of cost, there is no compromise in the performance efficiency [12]. And hence in this study, MySQL database has been opted and used to organize Tamil words and their corresponding details.

The initial phase in developing a database is to design the structure of the database. We used MySQL Workbench to model the DB structure. MySQL Workbench is a database design tool which helps in visually integrating SQL development, database creation, design and maintenance into a single Development Environment for the MySQL system [21]. MySQL Workbench provides excellent visual and technological tools that support database designers to do their jobs efficiently [22].

**Fig. 1:**
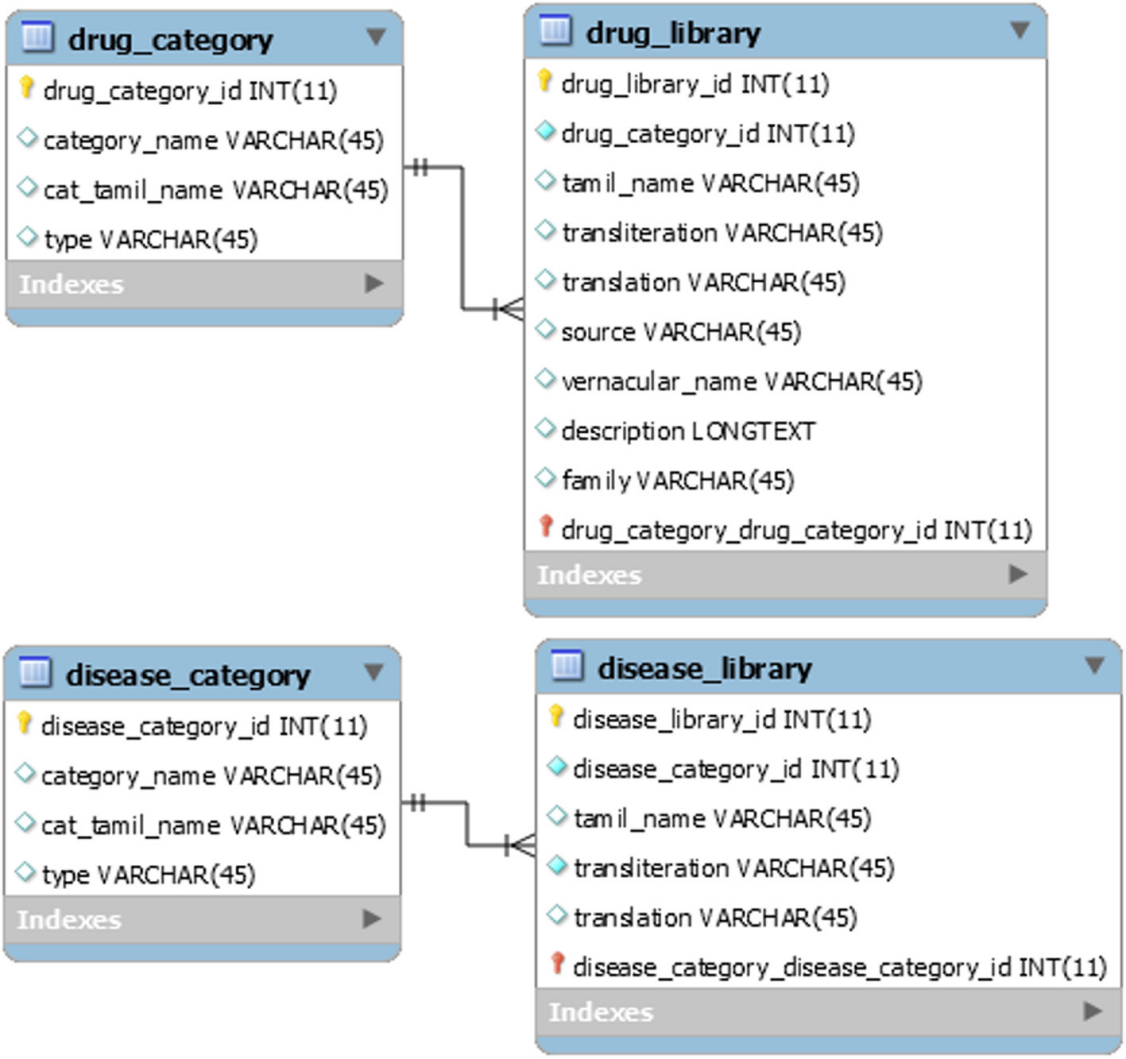
Drugs, disease category and library tables.

It is expected that every Siddha poem has information about any one of the details which includes diagnosis of disease, drug ingredients, products for treatment and modes of the treatment for specific diseases. Some poems have the combination of these details. Names of the diseases and drugs are extracted from the translated poems and then it is categorized into subsections. Separate tables for category of drugs and diseases are created with category id and category name. Then, tables for the drugs and disease library are created and linked with their corresponding category. Fig. 1 shows the design structure of drugs, disease category and library table created in the workbench.

**Fig. 2:**
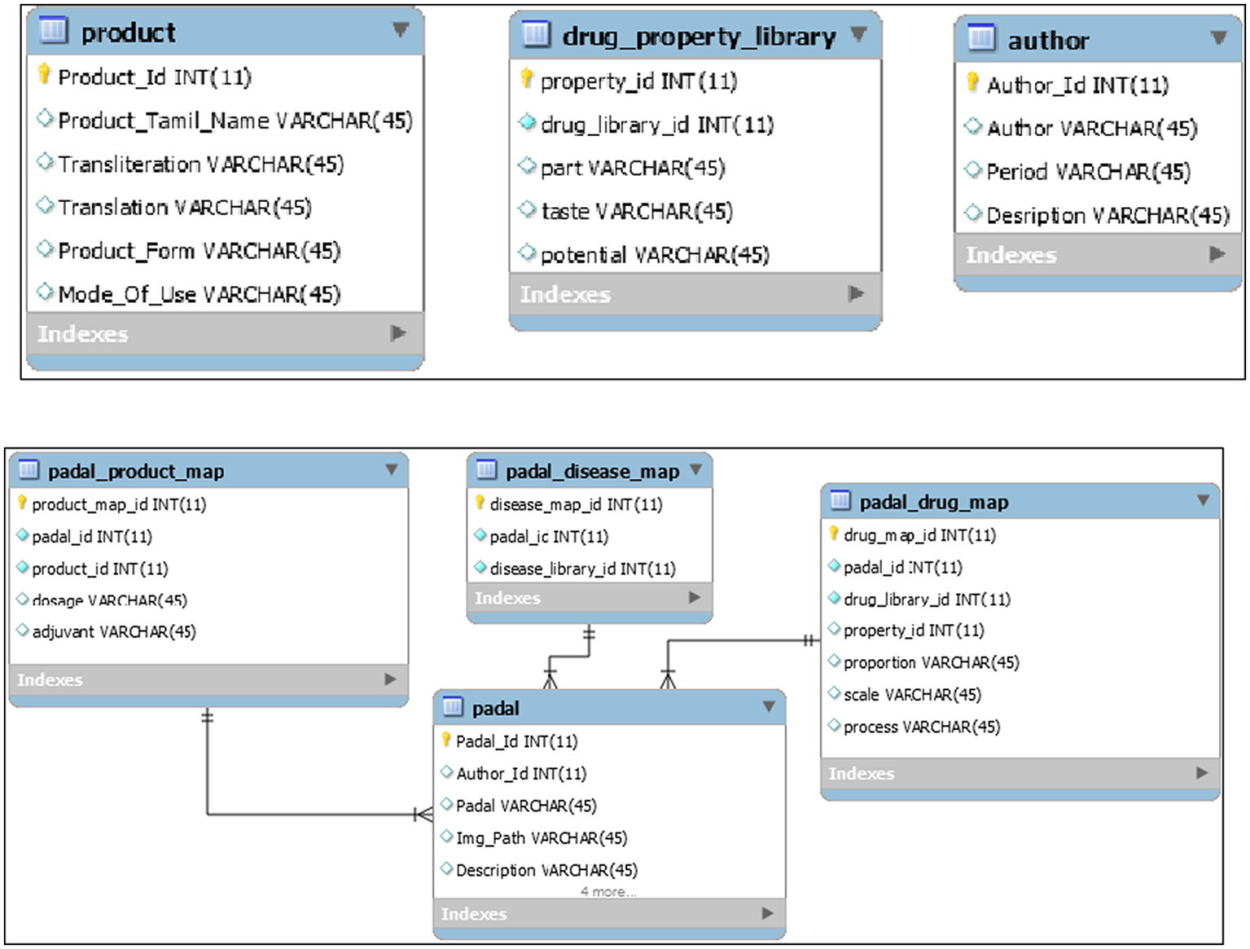
Product, drug, property and author tables.

Information on properties of drugs, products in the poem, author of the poem, and other details of Siddha poem are added to the separate table created for it. The table of products include the names of products, mode of use and form of the product. Properties of the drugs include part used, taste and potential of the drug. Author table includes the name of the author and the period of the author. The structure of the product, drug property, and author table is shown below in Fig. 2.

There is a separate table to store links of the Siddha poem and their authors, which is created as the table of poems (padal) and that table is mapped with the product, disease and drug using poem id and corresponding library id. Fig. 2 shows the structure of padal, padal_product_map, padal_disease_map and padal_drug_map tables.

**Fig. 3:**
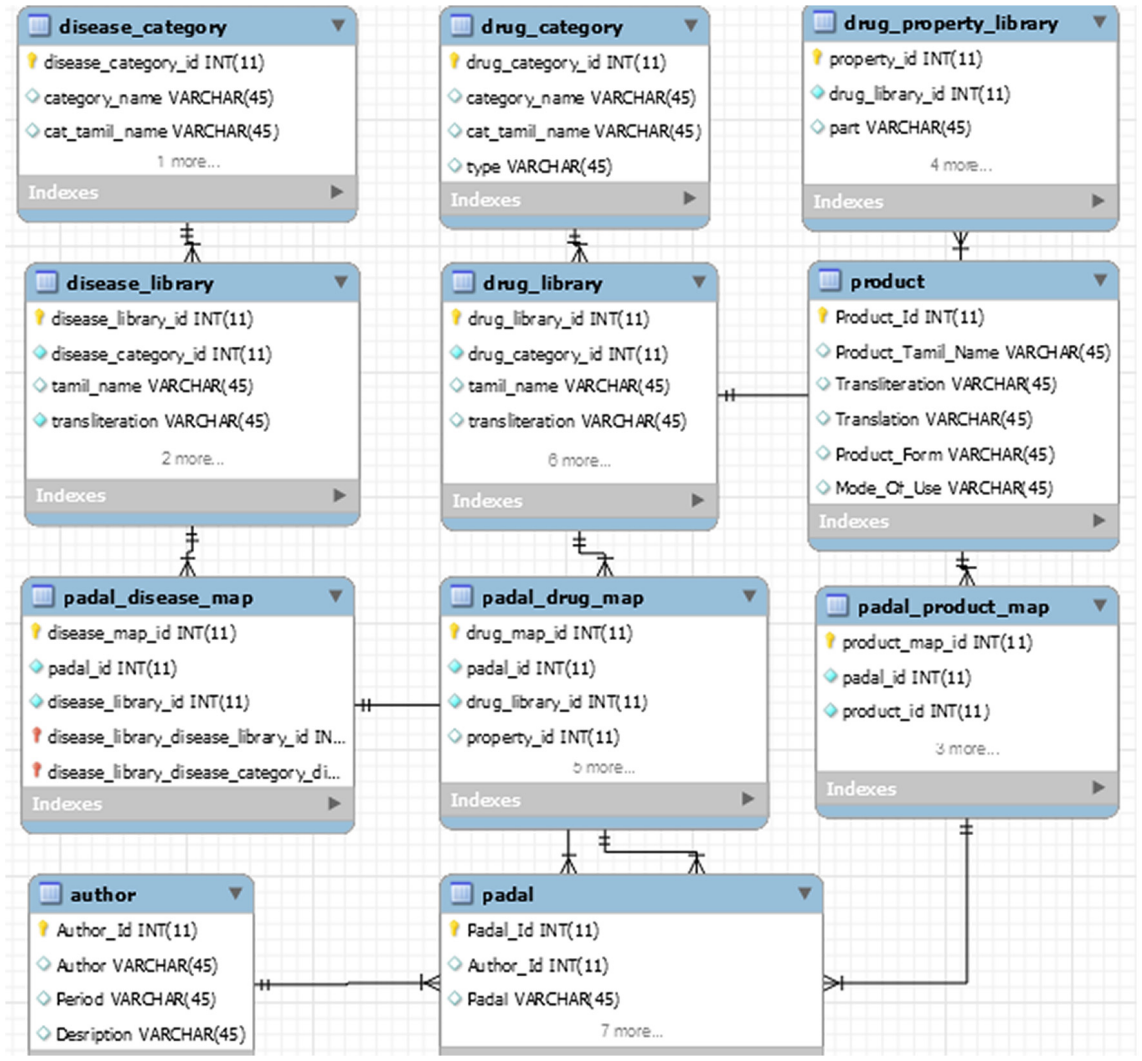
EER model.

The enhanced entity-relationship (EER) model (or extended entity-relationship model) is created in EER Diagram editor of MySQL Workbench. The EER diagram is developed to reflect more precisely the properties and constraints that are found in the database. Fig. 3 shows the screenshot of Siddha EER model for the Database Designed.

### 2.3 Webpages

Once the data library is created in the database, the next step is to create webpages. As of now, we have added 65 Siddha poems to the DB. HTML5, a markup language, is used for structuring and presenting the content on the webpage. *Home* page and *About Siddha* pages are developed as static Web Pages which are an introduction to Siddha medical system and details of this project. CSS (Cascading Style Sheets) is used to define the visual style of the web pages developed in HTML. *Inventory* and *Analytics* pages are developed as dynamic web pages where scripting language JavaScript is used to communicate with MySQL. Data is exchanged between browser and database as JSON (JavaScript Object Notation) data, a lightweight data-interchange format https://siddha.srmist.edu.in/index.html.

#### 2.3.1 Inventory page

Inventory page is the dynamic webpage, where the user has the option to select the author, disease, drug ingredient, and product from the available list. Users, on selecting the options available, corresponding Siddha poems and their details will be displayed in the form of a table. Disease, Ingredient, and product lists are loaded in the webpage from MySQL library tables as JSON data.

Based on the user options, the related details of the Siddha poem will be acquired from the DB and displayed on the table.

Tabulator.js, a jquery plugin is used to create tables and customized according to our need.

#### 2.3.2 Analytical page

Analytical page is developed to visualize the results (dashboard) which helps Siddha research and development team to understand or analyse the following for a particular disease,

• Treatment or products provided by various authors (Inter analysis) and multiple products suggested by the same author (Intra analysis)
• Potency based analysis on products
• Different taste of products available from different poems
• Kind of ingredients used and most commonly used ingredients across different product

Chart.js, an external JavaScript tool is used for Graphical representation by the chart to ease the analysis. Comparison between the poems of same authors and comparison of poems between the different authors are done to review the ingredients used for the same disease.

## 3 Results

**Fig. 4:**
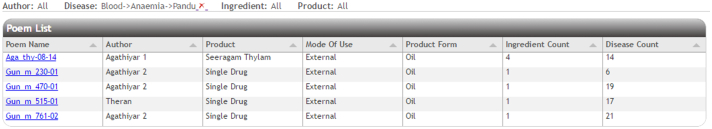
The list of poem for the disease selected.

This pilot design captured 110 medicinal preparations from two *Siddhars* (*Agathiyar and Theran*) and annotated. The generated annotations were indexed into the data repository created in eSS. The system can compare and visualize individual and multiple prescriptions to generate a hypothesis for siddha practitioners and researchers. In the inventory page, once the user selects disease/ingredient/product, corresponding poem and its details from DB gets listed in the table. The table has details of the poem which includes author, product, mode of use and form of the product, ingredient and disease count listed in the poem. Option selected by the user is displayed above the table. If the user selects the disease anaemia, then the table will show the list of poems related to anaemia that is available in the database as in Fig. 4.

**Fig. 5:**
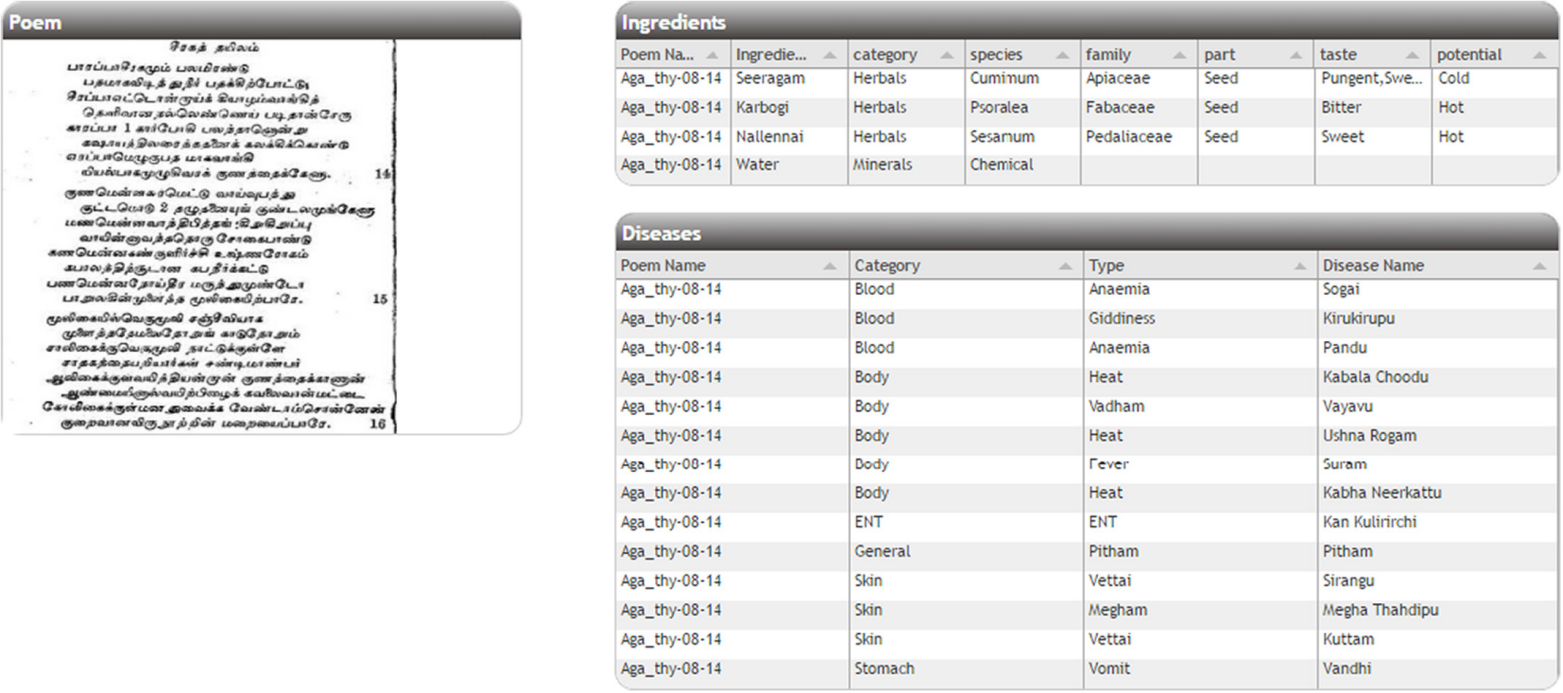
Selected poem, ingredients and disease table.

Link for the poem is provided in the first column of the table. Once the user clicks the link, details of the disease and the ingredients are extracted from the DB and displayed as a table in the webpage. Fig. 5 shows the screenshot of the selected poem and its details.

**Fig. 6:**
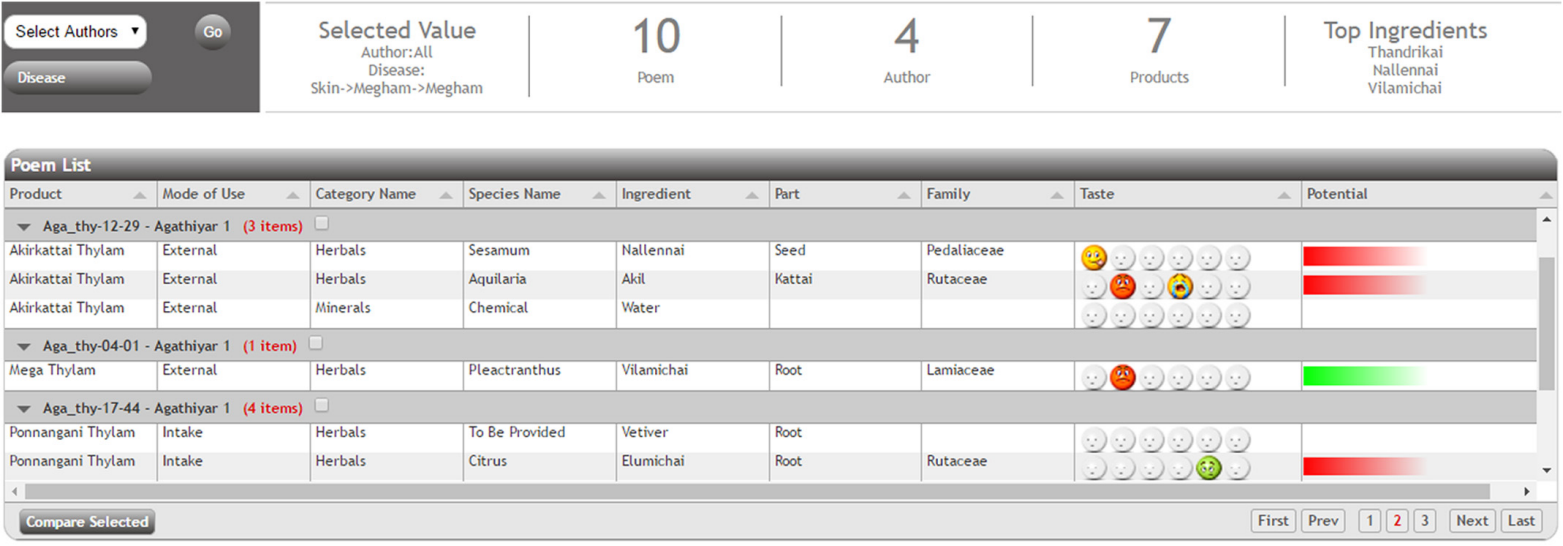
Analytics page table.

For example, in the Analytics page, once the user selects the disease like anaemia, the table with the list of poems related to anaemia and the details which include the ingredients used and properties of ingredients (taste, potential, family, and part) will be displayed. Selected value, number of poems, author and products will be displayed on the top of the table. The taste of the ingredient is indicated as smileys. The hot potential ingredient is indicated as red and the cold potential as green. For example, when the user selects the skin disease “Megham” all the available 10 poems in the database related to that disease will be listed in the table as shown in Fig. 6. The ingredients specified in the poem and their properties are also available in the table.

**Fig. 7:**
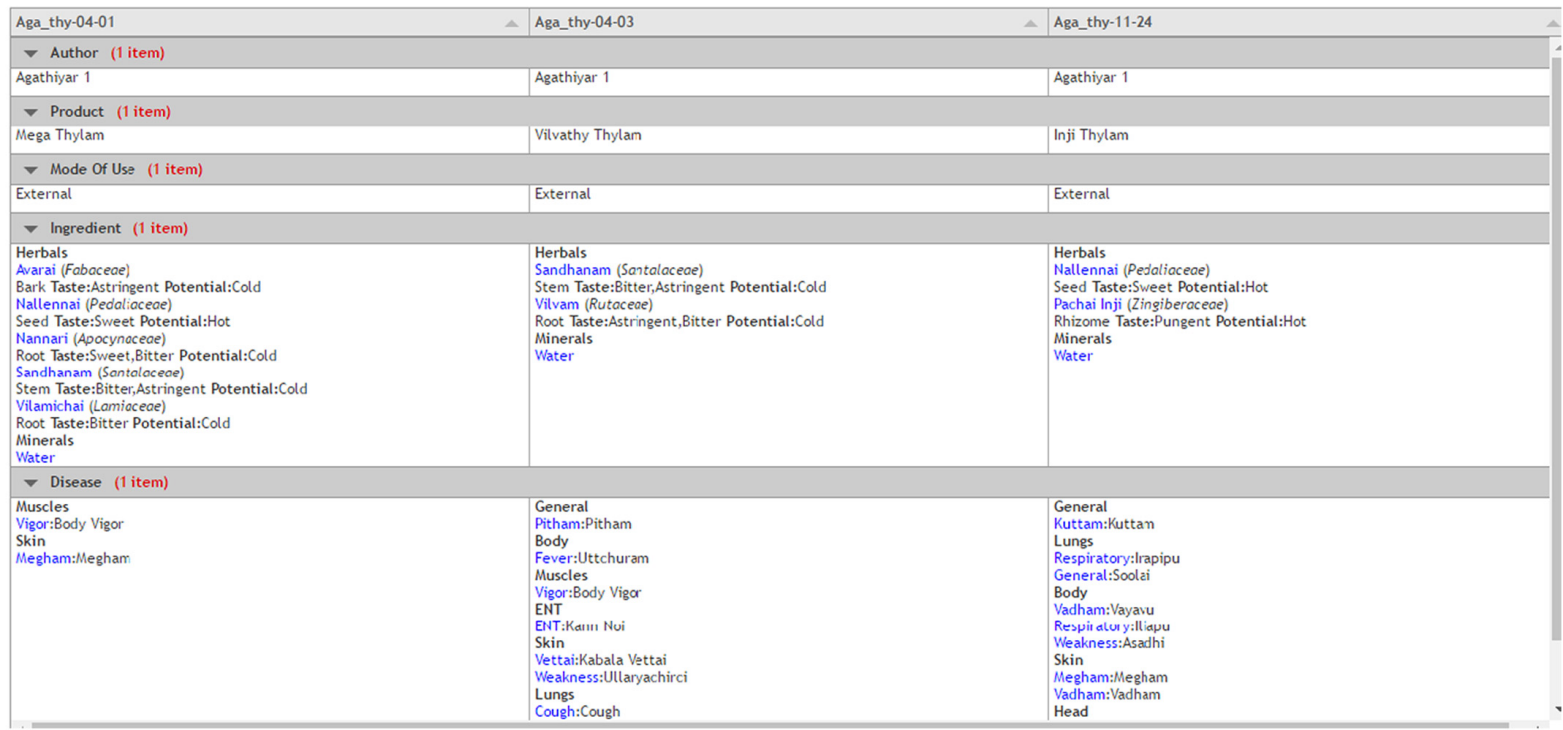
Compare page.

There is a “compare selected” button on the bottom of the table. If the user wants to compare any two or three poems for a selected disease, there is an option to check the poem and compare. A separate page will be opened with the comparison of selected poems. The comparison is based on the author, the product from the poems, mode of use, the ingredient used, and the disease specified in the poems. Fig. 7 shows the screenshot of the comparison page.

**Fig. 8:**
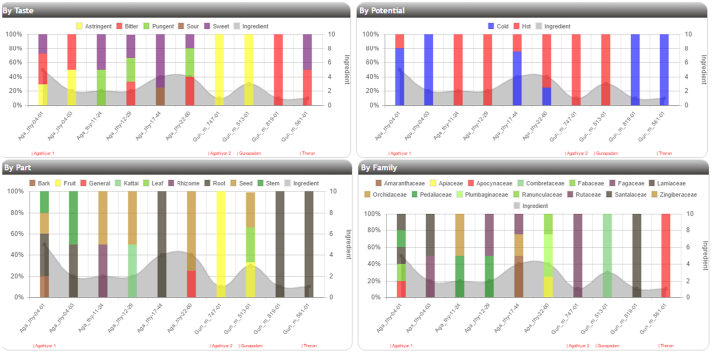
Analytics page comparison charts.

For a selected disease, the properties of the ingredients in those poems, like taste, potential, parts used and family of herbs usd will be compared between the same and different authors in the form of charts. Fig. 8 shows the screenshot of the comparison chart.

## 4 Discussions

Globally there are lots of advances in science, technology, and medicine; however quality healthcare to everybody is still not achieved. Unification of evidence based Traditional medicine with clinical practice will surely help in promoting health and to provide proper healthcare to everyone [1]. Siddha system of medicine is one of the most complete and distinguished traditional medicines, with a history of several thousand years of studying, practicing the diagnosis and treatment of, human and animal diseases. The Government of India has now digitized these materials to preserve the heritage of our country [23]. Siddha literature obtained from the historical period and from modern clinical studies has recently been transformed into digital data (translating/transcribing Siddha text and developing correlations with biological sciences) by the Literary Research & Documentation Department under the Central Council for Research in Siddha(CCRS).

Further this needs to be analyzed to find patterns and to discover some hidden secrets behind Siddhar's preparation and prescription of medicines and also facilitate research and development into knowledge discovery approaches and to modernize Siddha system of medicine. Researchers are not able to get adequate knowledge and information on Siddha drugs due to the language barrier. This study will help break the language barrier and make the Siddha content available globally. From the literature, it is evident that developed Traditional Chinese Medicine (TCM) databases assist to resolve various problems, which include extracting meaningful entity relation from TCM literature [24], drug discovery [25], treatment [26], understanding of TCM in Western countries and bridging the gap between traditional and modern system of medicine. Similarly a system termed Ayusoft is available for the traditional medicine Ayurveda that is developed to mine information from Ayurvedic texts and store it in knowledge repositories [27]. The system also supports analysis of information on classical Ayurvedic texts [[28], [29], [30]].

Systematically documenting and retrieving information of Siddha Medicine of the system is the pioneering effort. To our knowledge analytical exploration of the Siddha content has never been done before. Siddha Medicine system has innumerable number of poems which need to be uploaded and updated in the database. Webpage developed will be used to analytically explore the logic underlying the in-numerous drug formulations used by different Siddha authors, based on the factors like taste, potency, part and family of ingredients. And also the analytics system provides an opportunity to explore medicinal combinations which could be a source for developing time-tested novel treatment protocols. The future scope of project includes

• To expand the scope of analysis using various machine learning techniques like Text mining, supervised and unsupervised learning methods.
• The webpage will be made user-friendly and will serve for cross-language clients (Researchers & general Community)
• Admin page will be developed, where new siddha poems can be added by the users which will be validated by an expert to be updated in the database library.
• Authentication to access based on user roles.
• Gives opportunity for Systematic retrieval of Information related to Siddha pathology, pharmacology and treatment regimens.
• To explore medicinal combinations based on taste, potency, etc. to develop treatment protocols and trials.

## Declaration of Competin Interest

None.

## Appendix A Supplementary data

Supplementary material Image 1
