# Supplementary material for: Development of database structure and indexing for siddha medicine system – A platform for siddha literature analytics
Source: Dialogues Health. 2022 May 5;1:100008. doi: 10.1016/j.dialog.2022.100008 (PMC10953876; doi:10.1016/j.dialog.2022.100008)
Supplement: Supplementary file 1 — Supplementary material [file mmc1.docx]

1. Sample MySQL query to load the name of drugs from the database.

"SELECT A.category_name, A.Type,B.transliteration,B.drug_library_id FROM drug_category A,drug_library B WHERE A.drug_category_id = B.drug_category_id ORDER by 1,2,3"

1. Sample MySQL query to get the details of the poem for the disease selected.

"SELECT distinct P.PADAL_ID,P.PADAL,DIC.category_name,DIC.type,DIL.transliterationFROM padal P,Author A,padal_disease_map DIM, padal_drug_map dm,padal_product_map PM, disease_category DIC JOIN disease_library DIL USING (disease_category_id) WHERE A.Author_Id = P.Author_Id AND P.PADAL_ID = DM.Padal_Id AND P.padal_id = DIM.padal_id AND DIL.disease_library_id = DIM.disease_library_id AND P.PADAL_ID = PM.Padal_Id"
